# Oxytocin: taking the heat

**DOI:** 10.9745/GHSP-D-14-00102

**Published:** 2014-08-31

**Authors:** Stephen Hodgins

**Affiliations:** a Global Health: Science and Practice, Associate Editor for Maternal and Child Health

## Abstract

Oxytocin-in-Uniject satisfied the standards of its temperature-time indicator (TTI) in severe home storage conditions, although that required resupply every 30 days—a logistically onerous programmatic standard. Possible advances include: (1) incorporating TTIs with packaged batches of less expensive and more widely used conventional vials of oxytocin; (2) using TTIs calibrated more closely to the actual temperature sensitivity of oxytocin; and (3) researching whether a lower dose of oxytocin would be equally efficacious in preventing postpartum hemorrhage.

Globally, the leading cause of maternal deaths is postpartum hemorrhage (PPH). The single most important preventive intervention for PPH is active management of the third stage of labor, the most important element of which is administration of oxytocin (misoprostol, an orally administered drug, is almost as efficacious). It is encouraging that oxytocin now appears to be widely used for this purpose.^1^ Nevertheless, as Smith,^2^ in this issue of *Global Health: Science and Practice* (GHSP), points out, “challenges remain with … distribution, proper storage, and … maintaining a regular supply of the medicine.” There are reasons, in particular, to be concerned about *temperature stability* and *storage conditions*.

As with any pharmacologic intervention, an effective dose of the active ingredient needs to be delivered to the patient. The currently available formulations of oxytocin degrade more rapidly when exposed to high temperatures.^3^ In principle, in settings with high ambient temperatures, if a “cool-chain” (i.e., protection from high temperatures) cannot be maintained, we run the risk of administering an inadequate dose. Mullany and colleagues,^4^ in this issue of GHSP, make a useful contribution by raising this important issue in their drug storage simulation study in rural Ghana. Their approach is creative, the analysis sound, and the argument engaging.

In the study, the product used was Oxytocin-in-Uniject (OIU), delivering 10 International Units (IU). It should be noted that OIU is not in large-scale commercial production and costs at least 3 times as much as the conventional product. The standard form for PPH prevention is glass vials of 5 or 10 IU, at unit costs as low as US$0.10. In some markets, these vials are generally sold in blister packs.

The temperature-time indicator (TTI) (manufactured by the TempTime Corporation) currently incorporated as an element of the OIU device was used to determine the reject threshold. As the authors explain, the specific TTI used for the OIU is calibrated quite conservatively, resulting in the rejection of a considerable portion of oxytocin doses while they still fell within the industry-standard 90%–110% dosage specification band. Furthermore, as the authors also acknowledge, the Cochrane review^5^ of oxytocin efficacy for preventing PPH includes studies with doses ranging from 10 IU down to 3 IU, without any indication of the lower dose being any less effective in reducing blood loss (although no head-to-head comparison studies have yet been done).

Based on their findings, Mullany and colleagues conclude that even without special provisions for refrigeration at point-of-use, a stock resupply protocol that—on a *monthly* basis—recovers and disposes of any unused oxytocin would result in very few doses falling below the TTI reject threshold. But the trade-off here is achievement of high sensitivity for inefficacious product at the cost of low specificity; many doses that could still be clinically effective are likely to be rejected. More important is that full monthly replacement with new stock—with high reliability—may be quite challenging in many high-need settings, where logistics systems are often not very strong.

Much more widespread use of a temperature-time monitor for oxytocin, like the TTI currently used for OIU, would certainly be helpful in providing managers and clinicians a clear indication of which doses to reject and where there may be breaches in the “cool-chain.”

What might be some helpful next steps? We offer the following suggestions:

- In principle, TTIs could be incorporated in the packaging for *conventional* glass vials of oxytocin (for example, affixing them to blister-pack flats). At a cost of about 7 cents each, affixing one TTI to a blister-pack flat containing 8 or 10, 10 IU-vials would add less than 10% to the unit cost.
- To avoid unnecessary wastage, TTIs calibrated to more closely approximate the temperature sensitivity of oxytocin should be used.
- Rejecting a dose of oxytocin because it has slipped below the equivalent of 9 IU may be unnecessarily conservative; further dose-finding research could be warranted to determine whether a lower dose (say, 5 IU) would be equally efficacious for PPH prevention.

Oxytocin is an important weapon in our armamentarium for the reduction of PPH mortality. But to be effective, it needs to be protected against exposure to high temperatures. Considerable efforts have been made over the past decade to ensure that even women living in more remote areas benefit from giving birth with skilled health care providers. We need to ensure that these health care workers have all that is required to provide life-saving care, including efficacious medicines.
